# Prevalence of migraine subtypes in women with endometriosis and/or dysmenorrhea: results from a cross-sectional multicenter trial

**DOI:** 10.1186/s12905-026-04335-z

**Published:** 2026-02-13

**Authors:** Anna Cirkel, Hartmut Göbel, Carl Göbel, Ibrahim Alkatout, Antonia Kaiser, Norbert Brüggemann, Jens Minnerup, Achim Rody, Christoph Cirkel

**Affiliations:** 1 Department of Neurology, University Hospital Schleswig Holstein, Campus Luebeck, Ratzeburger Allee 160, Luebeck, Germany; 2 Kiel Migraine and Headache Centre, Kiel, Germany; 3 Department of Gynecology and Obstetrics, University Hospital Schleswig Holstein, Campus Kiel, Luebeck, Germany; 4 Department of Gynecology and Obstetrics, University Hospital Schleswig-Holstein, Campus Lübeck, Ratzeburger-Allee 160, Lübeck, 23538 Germany

**Keywords:** Endometriosis, Migraine, Migraine with aura, Menstrual migraine, Pure menstrual migraine, Dienogest.

## Abstract

**Background:**

Migraine and endometriosis are both debilitating chronic pain conditions that frequently co-occur in women. We aimed to assess the prevalence of migraine in women with surgically confirmed endometriosis (SCE) compared to women without surgically confirmed endometriosis (non-SCE), and to explore the distribution of migraine subtypes in this population.

**Methods:**

In a cross-sectional multicenter study, 838 women were recruited from two endometriosis centers and a patient association. Participants completed a standardized online questionnaire assessing migraine phenotype (ICHD-3 criteria), endometriosis status (surgically confirmed or not), and current hormone therapy. Group comparisons were performed using statistical tests, and odds ratios (OR) with 95% confidence intervals (CI) were calculated.

**Results:**

Surgically confirmed endometriosis (SCE) was present in 561 participants (67%), whereas 277 (33%) had no surgical confirmation (non-SCE). A total of 320 (38.2%) participants reported migraine. Migraine was more prevalent in women with SCE than in those without (41.0% vs. 32.5%; OR 1.44, 95% CI 1.07–1.95), and remained associated after adjustment for age, BMI, education, and hormonal therapy (adjusted OR 1.38, *p* = 0.048). Among those with migraine, 204 (63.8%) had non-menstrual migraine (NM), 93 (29.1%) had menstrually-related migraine (RM) and 23 (7.2%) had pure menstrual migraine (PM). Migraine with aura showed higher odds in the SCE group (OR 1.61, 95% CI 1.11–2.35), as did PM (OR 11.27, 95% CI 1.51–84.01). RM was associated with higher migraine frequency (*p* < 0.001), pain intensity (*p* = 0.019), and greater functional impairment (*p* < 0.001) than NM or PM. Participants with PM had lower rates of endocrine endometriosis/dysmenorrhea treatments (17.4% vs. 54.4% (NM) vs. 34.4% (RM); *p* < 0.001).

**Conclusion:**

Women with SCE show a higher prevalence of migraine – particularly migraine with aura and PM – than those without SCE. The observed co-occurrence of endometriosis and migraine highlights the need for increased clinical awareness and further prospective studies to explore implications for interdisciplinary management. However, no causality can be inferred from this cross-sectional study.

**Supplementary Information:**

The online version contains supplementary material available at 10.1186/s12905-026-04335-z.

## Background

 Endometriosis and migraine are both chronic pain disorders that frequently occur in women and may significantly reduce their quality of life. Migraine is a complex neurological condition characterized by recurrent headaches, accompanied by nausea, vomiting, and sensory hypersensitivity [1]. Endometriosis, in contrast, is a chronic inflammatory disease presenting with endometrium-like glands and stroma surrounded by cytogenic stroma outside the uterus, mainly affecting premenopausal women [2]. Although these conditions involve a variety of organ systems, increasing evidence suggests a strong mutual association between them, pointing to potential shared pathophysiological mechanisms [3]. The exact mechanisms underlying this association remain unclear. However, hormonal fluctuations, neuroinflammatory processes, and central sensitization are believed to play a role. Both disorders are highly dependent on estrogen levels, and dysregulated estrogen signaling has been implicated in pain modulation within the central nervous system [4].

The exact prevalence of endometriosis is unknown. However, estimates range from 2% to 10% of women [2]. Typical endometriosis symptoms are dysmenorrhea, pelvic pain, infertility, dyspareunia, urinary tract and bowel symptoms [5]. Endometriosis may also be asymptomatic [6, 7].The symptoms have the potential to impact quality of life, physical abilities, everyday activities, social life, as well as work, sexual relations and psychological health [2, 8]. Ultrasonography may indicate the presence of endometriosis in some cases [2]. Typically, endometriosis is diagnosed by histopathological investigation. Obtaining a specimen for histopathology is accompanied by the simultaneous removal of endometriotic lesions. Symptomatic endometriosis – especially pain – is typically treated with surgical removal of endometriosis lesions, endocrine medication, non-medical strategies, or combinations of these [2]. In the case of Dysmenorrhea may also be effectively treated with non-steroidal anti-inflammatory drugs. Endocrine treatment options involve progestogens, combined hormonal contraceptives, GnRH agonists, or GnRH antagonists [2].

Migraine is characterized by typical symptoms, including moderate to severe headache, nausea, vomiting, and light or sound hypersensitivity. The global age-standardized prevalence of migraine is 14.4%. However, the condition is clearly age and sex related; in other words, it affects women more frequently than men and the age peak is 35–39 years [1]. According to the International Classification of Headache Disorders, migraine attacks may occur with or without an aura [9]. The appendix further subclassifies migraine into pure menstrual migraine (PM), menstrually-related migraine (RM), and non-menstrual migraine (NM) [9]. Clearly, the subclassification is only applicable for menstruating women [9]. PM exclusively occurs “on day 1 ± 2 (i.e., days − 2 to + 3) of menstruation in at least two out of three menstrual cycles and at no other times of the cycle”. The first day of menstruation counts as day 1, the preceding day is day − 1 (there is no day 0). RM may occur at other times of the cycle as well. NM does neither fulfil the criteria for pure menstrual migraine nor for menstrually-related migraine [9]. Hormone prophylaxis (continuous intake, ideally suppressing menstruation) is known to be more effective in PM than in RM [9, 10]. The continuous intake of progestin-only pills reduces the number of migraine attacks and the number of migraine days per month [11]. A variety of hypotheses have been proposed for the presence of PM and RM, including estrogen withdrawal and genetic aspects [12]. Like endometriosis, migraine may also affect work and family life, as well as social activities [1].

Understanding the interplay between migraine and endometriosis—particularly their shared hormonal and pain-modulatory mechanisms—is clinically relevant, as it could lead to tailored therapeutic strategies that may alleviate symptoms in both conditions. Addressing the overlap between these two chronic pain disorders may also improve clinical recognition and interdisciplinary management between neurology and gynecology.

Previous research has been mainly focused on observational data indicating a correlation between migraine and endometriosis, yet studies that differentiate migraine subtypes within this population are scarce [13, 14]. Based on interdisciplinary cooperation between neurologists and gynecologists, the present study provides a novel and thorough overview of the migraine subtype co-occurrence in women with and without surgically confirmed endometriosis.

We hypothesized that women with surgically confirmed endometriosis (SCE) have a higher overall prevalence of migraine compared to women without surgically confirmed endometriosis (non-SCE).

The primary objective of this study was to determine the prevalence of migraine in women with and without SCE. The secondary objectives were to (i) analyze the distribution of migraine subtypes (NM, RM, PM, with and without aura), (ii) assess their associated symptom burden, and (iii) evaluate patterns of hormonal treatment among affected and non-affected women with endometriosis.

## Methods

### Study design and population

The study was approved by the ethics committee of the University of Lübeck (AZ2023-287) before the recruitment of participants in 2023. All subjects consented to their participation and anonymized data storage. Participation in the study was anonymous and voluntary.

**Fig. 1:**
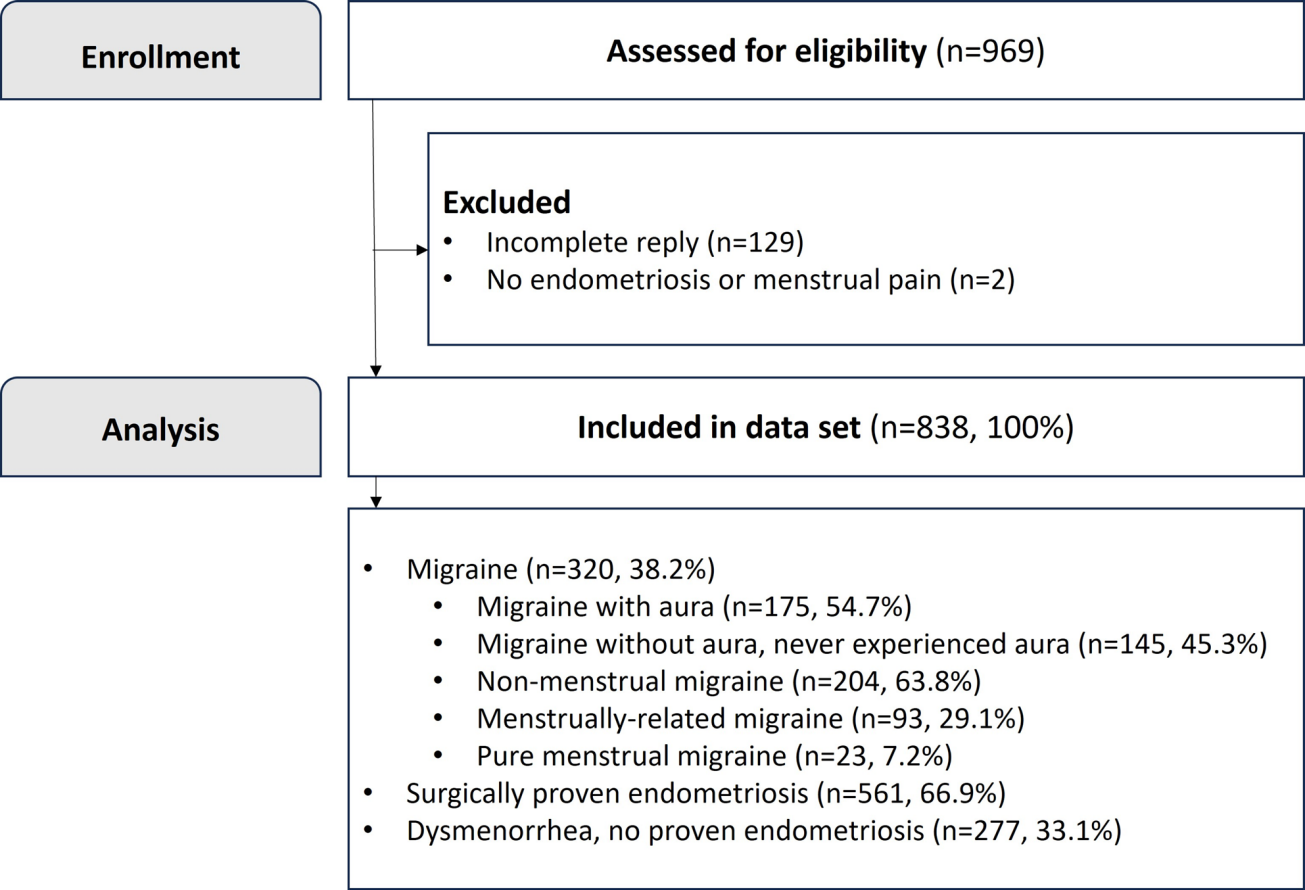
Flowchart of participant enrollment and analysis. A total of 813 email invitations were sent from the participating centers, of which 236 were completed (response rate 29%). The number of individuals reached via the Endometriosis Association’s homepage and social media could not be quantified

We used an online platform (www.umfrageonline.com) for the cross-sectional multicenter investigation. The online survey permitted participation from May to November 2023. Invitations were sent by mail in May 2023 at two endometriosis centers, from two campuses of a university hospital, to participants who had visited the facility from January 2017 to March 2023 and allowed email follow-up. Additional recruitment occurred with the support of the German Endometriosis Association via an online invitation on their homepage and social media. Inclusion criteria were the female gender, understanding the German survey, postmenarchal and premenopausal status, and a history of menstrual pain and/or endometriosis. Participants with incomplete questionnaires and those who did not fulfil the inclusion criteria were excluded (Fig. 1). Prior to completing the online questionnaire, all participants received written information about the average expected response time and study aims. The survey included very detailed questions regarding (1) sociodemographic and clinical data (age, BMI, education level), (2) migraine phenotype according to the IHS [9] (including frequency and pain intensity; all migraine subtypes were evaluated exactly according to the IHS criteria [9]), (3) migraine treatment (oral medication, medical consultation), (4) quality of life and the burden of migraine pain, (5) menstrual pain and other endometriosis symptoms (menstrual pain intensity, frequency, quantity of menstrual bleeding), (6) quality of life and the burden of dysmenorrhea/endometriosis, (7) surgical diagnosis (and treatment) of endometriosis, and (8) hormonal and pain treatment of dysmenorrhea/endometriosis (including the type of hormone treatment and regimen). The questionnaire was developed for this study and the English translation of questions from the questionnaire which have been analyzed are presented in the supplements.

The questionnaire was answered in total by 969 persons; 838 were included in the analysis (women with a mean age of 30.68 ± 6.92 years, range 15–54 years). Of the 813 email invitations sent, 236 complete responses were received (response rate 29%). The total number of individuals reached via social media and homepage could not be quantified. Therefore, a precise denominator for the total number of invitations received is unavailable. Further details are shown in Fig. 1.

### Statistical analysis

Data analysis was performed with the Statistical Package for Social Sciences (IBM SPSS Statistics for MAC, Version 22.0. Armonk, NY: IBM Corp), and statistical significance was set to *p* < 0.05 (two-sided). Group comparisons for continuous data were performed using Welch’s ANOVA (reported as F and p values). The Shapiro-Wilk test was performed to ascertain whether continuous variables were normally distributed. Associations between categorical variables were analyzed using Pearson’s Chi-square (χ²) test or Fisher’s exact test, as appropriate. Results are reported as χ² and corresponding p-values. Ordinal variables were analyzed using the Kruskal–Wallis test, and results are reported as H and p values. The association with migraine subtypes and surgically confirmed endometriosis (SCE) in comparison to participants with dysmenorrhea but without surgically confirmed endometriosis (non-SCE), was calculated using the crude odds ratio (OR) and the 95% confidence interval (CI). To confirm the robustness of the findings, a multivariable logistic regression adjusting for age, BMI, educational level, and current hormonal therapy was additionally conducted. The multivariable adjustment was applied for migraine overall, whereas odds ratios for migraine subtypes were reported as crude values. Results of the descriptive analysis were expressed as mean ± standard deviation (SD); absolute numbers of cases are also stated.

Given the exploratory nature of the study, no formal adjustment for multiple comparisons was applied. Therefore, p-values are interpreted descriptively.

## Results

### Comparison of migraine between women with and without surgically confirmed endometriosis (SCE)

A total of 838 women fulfilled the inclusion criteria, of whom 561 (67%) had surgically confirmed endometriosis (SCE) and 277 (33%) had dysmenorrhea without surgical confirmation of endometriosis (non-SCE).

Women with SCE were on average older (31.75 ± 6.88 years) than those without surgical confirmation (28.51 ± 6.50 years; *p* < 0.001) and had a higher BMI (25.31 ± 5.47 kg/m² vs. 23.75 ± 4.52 kg/m²; *p* < 0.001). They also showed a slightly lower educational level compared with non-SCE participants (*p* = 0.020) (see Table 1).

**Table 1:** Sociodemographic and clinical characteristics of all study participants with and without surgically confirmed endometriosis (SCE)

Variable		Unit	Participants with SCE		Participants with non-SCE		Statistics	
			*n*=561(100%)	Mean ± SD (95% CI) / median [IQR] / *n* (%)	*n*=277 (100%)	Mean ± SD (95% CI) / median [IQR] / *n* (%)	Test / Statistic	*p*-value
Sociodemographics								
Age		years	561	31.75 ± 6.88 (31.18 – 32.32)	277	28.51 ± 6.50 (27.74 – 29.28)	F=44.26	<0.001*
BMI		kg/m^2^	557	25.31 ± 5.47 (24.85 – 25.76)	277	23.75 ± 4.52 (23.21 – 24.28)	F=19.10	<0.001*
Educational level		^1^	560	3 [2–4]	277	3 [3–4]	H=5.40	0.020*
Migraine pain (any)								
Frequency of migraine attacks		Every few months / Repeatedly per month	230	124 (72.9 %) / 106 (71.1 %)	89	46 (27.1 %) / 43 (28.9 %)	χ² = 0.13	>0.05
		days/month	106	4.48 ± 3.46 (3.82 – 5.15)	43	4.72± 4.50 (3.34 – 6.11)	F=0.10	>0.05
Intensity of migraine pain		VAS 0-10	230	7.24 ± 1.48 (7.05 – 7.44)	90	7.16 ± 1.59 (6.82 – 7.49)	F=0.21	>0.05
Frequency of pain medication use		days/month	215	2.93 ± 3.35 (2.48 – 3.38)	85	3.60 ± 4.33 (2.67 – 4.53)	F=1.67	>0.05
Restriction in job activities		^2^	229	2 [2–3]	90	2 [2–3]	H=0.121	>0.05
Restriction in leisure activities		^2^	229	2 [2–3]	90	2 [2–3]	H=0.260	>0.05
Restriction in family activities		^2^	230	2 [2–3]	90	2 [1–3]	H=0.005	>0.05
Menstrual pain								
Quantity of menstrual bleeding		^3^	561	3 [2 – 3]	276	3 [2–3]	H=5.48	0.02*^+^
VAS score during the last 3 months		VAS 0-10	485	7.26 ± 1.97 (7.06 – 7.45)	243	7.40 ± 1.69 (7.18 – 7.62)	F=0.90	>0.05
Disturbance of dysmenorrhea		^4^	553	4 [4–5]	275	4 [4–5]	H=0.70	>0.05
Frequency of pain medication intake		^5^	472	3 [2–3]	233	3 [2–3]	H=0.02	>0.05
VAS score under pain medication treatment		VAS 0-10	485	5.15 (4.93 – 5.36)	243	4.82 (4.54 – 5.10)	F=3.39	>0.05
Restriction in job activities		^2^	560	3 [2–4]	274	3 [2–3]	H=1.78	>0.05
Restriction in leisure activities		^2^		3 [2–3]	276	3 [2–3]	H=0.17	>0.05
Restriction in family activities		^2^	559	3 [2–3]	275	2 [2–3]	H=1.16	>0.05
Present intake of hormone treatment (contraceptive pill/endocrine dysmenorrhea/endometriosis treatment)								
Intake yes or no		Yes (%)	561	275 (49 %)	277	95 (34.3 %)	χ²= 16.30	<0.001*
Known progestin-only vs. estrogen-containing		Progestin only (%)	275	207 (75.3 %)	94	53 (56.4 %)	χ²= 12.01	<0.001*
Intake regimen	Continuous	Yes (%)	275	222 (80.7 %)	95	64 (67.4 %)	χ²= 7.18	0.007*
	With break/placebo interval			7 (2.5 %)		14 (14.7 %)	χ²= 19.60	<0.001*
	IUD			44 (16 %)		16 ( 16.8 %)	χ²= 0.04	>0.05

Data are presented as mean ± SD (95 % CI) for continuous variables, median [IQR] for ordinal variables, and n ( %) for categorical variables. Statistical tests used: Welch’s ANOVA for continuous variables, χ²-test for categorical variables, and Kruskal–Wallis-test for ordinal variables

^1^0= no school leaving certificate, secondary school (Hauptschule) = 1, intermediate school (Mittlere Reife) = 2, high school (Abitur/Fachabitur) = 3, university degree = 4

^2^not at all = 0, slightly = 1, moderately = 2, severely = 3, very severely = 4

^3^no bleeding = 0, light bleeding = 1, moderate bleeding = 2, strong bleeding = 3

^4^no menstrual pain = 0, not disturbing = 1, marginally disturbing = 2, moderately disturbing = 3, severely disturbing = 4, very severely disturbing = 5

^5^0=never, 1=in less than 2 of 3 menstruations, 2=in two of three menstruations, 3=in every menstruation

^6^known gestagen-only ingredient = 1, known estrogen/gestagen ingredient = 2

^+^Higher mean ranks indicate stronger menstrual bleeding in the SCE group

*Significant results are highlighted in bold

Overall, 320 women (38.2%) reported a migraine diagnosis. The prevalence of migraine (any type) was higher among women with SCE compared to those without SCE (41.0% vs. 32.5%; *p* = 0.04). The crude odds ratio for any migraine diagnosis in SCE patients was 1.44 (95% CI 1.07–1.95). In multivariable analyses adjusting for age, BMI, education, and current hormonal treatment, surgically confirmed endometriosis (SCE) remained associated with migraine overall (adjusted OR 1.38, 95% CI 1.00–1.89, *p* = 0.048) (Fig. 3).

Analysis for migraine subtypes revealed that the crude odds ratio of migraine with aura (OR 1.61, 95%CI 1.11–2.35), and the crude odds ratio of pure menstrual migraine (OR 11.27, 95%CI 1.51–84.01; Fig. 3) were higher in patients with SCE. The odds ratios of further subtypes (migrainewith no history of aura, menstrually-related migraine, non-menstrual migraine) were not increased (Fig. 3).

Within the non-SCE group, migraine prevalence did not differ significantly between women with and without prior surgery (35.8% vs. 32.6%; χ² = 0.16, *p* = 0.69). In the adjusted model, the association remained non-significant (aOR 0.77 [95% CI 0.36–1.63], *p* = 0.49). These results confirm that the non-SCE subgroup can be considered internally homogeneous with respect to odds of migraine.

Women with SCE also reported heavier menstrual bleeding (median 3 [2–3] vs. 3 [2–3]; indicated by higher mean rank; *p* = 0.02) and similar mean menstrual pain intensity (VAS 7.26 ± 1.97 vs. 7.40 ± 1.69; *p* > 0.05). The disturbance level due to dysmenorrhea did not differ significantly between groups.

Regarding hormonal therapy, the current use of hormone treatment was more common among women with SCE (49.0%) than among those without (34.3%; *p* < 0.001). Among current users, progestin-only regimens predominated in both groups (75.3% vs. 56.4%; *p* < 0.001), and a continuous intake regimen was reported more frequently by women with SCE (80.7% vs. 67.4%; *p* = 0.007).

Among women without migraine, an association between surgical status and current hormone therapy use was observed, reaching statistical significance (*p* = 0.027),), with higher hormone therapy rates in the SCE group compared to non-SCE participants (Table 4).

Table 1 summarizes the sociodemographic and clinical characteristics of all study participants with and without surgically confirmed endometriosis.

### Comparison between women with endometriosis with and without migraine

Within the SCE group, 230 women reported migraine and 331 reported no migraine.

Women with and without migraine were comparable regarding age, BMI, and education (all *p* > 0.05).

However, women with migraine reported higher menstrual pain intensity (VAS 7.5 ± 1.7 vs. 7.1 ± 2.1; *p* = 0.02) and stronger menstrual bleeding (*p* = 0.02). They also showed greater restrictions in leisure (*p* = 0.01) and family activities (*p* < 0.001), but not regarding their job (*p* > 0.05).

Hormone therapy use and regimen type did not differ significantly between groups (all *p* > 0.05).

Table 2 summarizes the sociodemographic and clinical differences between endometriosis participants with and without migraine.

**Table 2:** Comparison between women with SCE with and without migraine

Variable		Unit	Participants with SCE and Migraine		Participants with SCE without Migraine		Statistics	
			*n*= 230 (100%)	Mean (95% CI) / *n* (%)	*n*= 331 (100%)	Mean (95% CI) / *n* (%)	Test / Statistic	*p*-value
Sociodemographics								
Age		years	230	31.72 ± 7.35 (30.76 – 32.67)	331	31.77± 6.54 (31.06 – 32.47)	F = 0.01	>0.05
BMI		kg/m^2^	228	25.72 ± 5.46 (25.01 – 26.43)	329	25.02 ± 5.47 (24.43 – 25.61)	F=2.22	>0.05
Educational level		^1^	230	3 [2 – 4]	330	3 [2 – 4]	H=1.301	>0.05
Menstrual pain								
Quantity of menstrual bleeding		^3^	230	3 [2 – 3]	331	2 [2 – 3]	H=5.939	0.02*
VAS score during the last 3 months		VAS 0-10	165	7.51 ± 1.69 (7.25 – 7.77)	229	7.07 ± 2.13 (6.80-7.35)	F=5.1	0.02*
Disturbance of menstruation		^4^	230	4 [4 – 5]	320	4 [4 – 5]	H=0.792	>0.05
Frequency of pain medication intake		^5^	197	3 [3 – 3]	275	3 [3 – 3]	H=0.848	>0.05
VAS score under pain medication treatment		VAS 0-10	209	5.34 ± 2.38 (5.01 – 5.66)	276	5.0 ± 2.39 (4.72 – 5.28)	F=2.41	>0.05
Restriction in job activities		^2^	230	3 [2 – 3]	320	3 [2 – 3]	H=2.404	>0.05
Restriction in leisure activities		^2^		3 [2 – 4]	320	3 [2 – 3]	H=6.090	0.014*
Restriction in family activities		^2^		3 [2 – 3]	320	2 [2 – 3]	H=11.251	<0.001*
Present intake of hormone treatment (contraceptive pill/endocrine dysmenorrhea/endometriosis treatment)								
Intake yes or no		Yes (%)	230	115 (50 %)	331	160 (48.3 %)	χ² = 0.15	>0.05
Known progestin-only vs. estrogen-containing		Progestin only (%)	115	87 (75.7 %)	160	120 (75 %)	χ²= 0.02	>0.05
Intake regimen	Continuous	Yes (%)	115	94 (81.7 %)	160	128 (80.0 %)	χ² = 0.13	>0.05
	With break/placebo interval		115	2 (1.7%)	160	5 (3.1%)	χ²= 0.52	>0.05
	IUD		115	19 (16.5 %)	160	25 (15.6 %)	χ² = 0.04	>0.05

Data include sociodemographic variables, menstrual pain intensity, endometriosis-related symptoms and current hormone therapy. Data are presented as mean ± SD (95 % CI) for continuous variables, median [IQR] for ordinal variables, and n (%) for categorical variables

Statistical tests: Welch’s ANOVA for continuous variables, χ² or Fisher’s exact tests for categorical variables, and Kruskal–Wallis tests for ordinal variables

^1^0= no school leaving certificate, secondary school (Hauptschule) = 1, intermediate school (Mittlere Reife) = 2, high school (Abitur/Fachabitur) = 3, university degree = 4

^2^not at all = 0, slightly = 1, moderately = 2, severely = 3, very severely = 4

^3^no bleeding = 0, light bleeding = 1, moderate bleeding = 2, strong bleeding = 3

^4^no menstrual pain = 0, not disturbing = 1, marginally disturbing = 2, moderately disturbing = 3, severely disturbing = 4, very severely disturbing = 5

^5^0=never, 1=in less than 2 of 3 menstruations, 2=in two of three menstruations, 3=in every menstruation

*Significant results are highlighted in bold

### Migraine subtype analyses

Among all women with migraine (*n* = 320), 204 (63.8%) had non-menstrual migraine (NM), 93 (29.1%) menstrually related migraine (RM), and 23 (7.2%) pure menstrual migraine (PM).

The mean intensity of migraine pain on the visual analog scale (0 = no pain, 10 = highest imaginable pain) exceeded 7 in all three groups, with the highest intensity in RM participants (*p* = 0.019; Table 3). The frequency of migraine attacks per month and associated functional restrictions in work, leisure and family life were also most pronounced in RM (*p* < 0.001; Tabel 3)..

**Table 3:** Participants Clinical and sociodemographic characteristics of women with migraine, stratified by migraine subtype: non-menstrual migraine (NM), menstrually-related migraine (RM), and pure menstrual migraine (PM)

Variable		Unit	Participants with non-menstrual migraine		Participants with menstrually-related migraine		Participants with pure menstrual migraine		Statistics	
			*n*=204 (100%)	Mean ± SD (95% CI) / median [IQR] / *n* (%)	*n*=93 (100%)	Mean ± SD (95% CI) / median [IQR] / *n* (%)	*n*=23 (100%)	Mean ± SD (95% CI) / median [IQR] / *n* (%)	Test / Statistic	*p*-value
Sociodemographics										
Age		years	204 (100%)	29.88 ± 7.09 (28.90 – 30.86)	93 (100%)	32.97 ± 7.25 (31.48 – 34.46)	23 (100%)	32.43 ± 6.97 (29.42 – 35.45)	F=6.38	0.003*
BMI		kg/m^2^	203 (99.5 %)	24.56 ± 4.82 (23.90 – 25.23)	92 (98.9%)	25.69 ± 5.42 (24.57 – 26.81)	23 (100%)	27.22 ± 5.87 (24.68 – 29.76)	F=3.19	*0.049*
Educational level		^1^	204 (100%)	3 [2–4]	92 (98.9%)	3 [3–4]	23 (100%)	4 [2–4]	H=1.89	>0.05
Migraine pain										
Frequency of migraine attacks		every few months / Repeatedly per month	203	127 (62.6 %) / 76 (37.4%)	93	27 (29.0 %) / 66 (71.0 %)	23	16 (69.6 %) / 7 (30.4 %)	χ²=31.44	<0.001*
		days/month	76 (37.3%)	4.66 ± 3.66 (3.82 – 5.49)	66 (71.0%)	4.65 ± 4.05 (3.66 – 5.65)	7 (30.4%)	2.43 ± 0.79 (1.70 – 3.16)	F=12.78	<0.001*
Intensity of migraine pain		VAS 0-10	204 (100%)	7.07 ± 1.49 (6.86 – 7.27)	93 (100%)	7.60 ± 1.51 (7.29 – 7.91)	23 (100%)	7.00 ± 1.48 (6.63 – 7.64)	F=4.26	0.019*
Frequency of pain medication use		days/month	187 (91.7%)	2.97 ± 4.03 (2.39 – 3.55)	91 (97.8%)	3.74 ± 3.14 (3.08 – 4.39)	22 (95.7%)	1.82 ± 0.91 (1.42 – 2.22)	F=14.45	<0.001*
Migraine aura present		yes		111 (54.4 %)		54 (58.1%)		10 (43.5%)	χ²=1.60	>0.05
Restriction in job activities		^2^	204 (100%)	2 [2–3]	92 (98.9%)	3 [2–4]	23 (100%)	2 [2–3]	H=26.11	<0.001*
Restriction in leisure activities		^2^	203 (99.5%)	2 [2–3]	93 (100%)	3 [2–4]	23 (100%)	2 [2–3]	H=18.04	<0.001*
Restriction in family activities		^2^	204 (100%)	2 [2–3]	93 (100%)	3 [2–4]	23 (100%)	2 [2–3]	H=23.22	<0.001*
Menstrual pain										
Quantity of menstrual bleeding		^3^	204 (100%)	2 [2–3]	93 (100%)	3 [2–3]	23 (100%)	3 [3–3]	H=8.42	0.015*
VAS score during the last 3 months		VAS 0-10	144 (70.6%)	7.48 ± 1.75 (7.19 – 7.77)	76 (81.7%)	7.46 ± 1.74 (7.06 – 7.86)	20 (87.0%)	7.70 ± 1.34 (7.07 – 8.33)	F=0.25	>0.05
Disturbance of Dysmenorrhea		^4^	204 (100%)	4 [4–5]	93 (100%)	4 [4–5]	23 (100%)	4 [4–5]	H=0.03	>0.05
Frequency of pain medication intake		^5^	174 (85.3%)	3 [3–3]	78 (83.9%)	3 [3–3]	21 (91.3%)	3 [2–3]	H=1.15	>0.05
VAS score under pain medication treatment		VAS 0-10	183 (89.7%)	5.08 ± 2.52 (4.71 – 5.44)	83 (89.2%)	5.51 ± 1.97 (5.08 – 5.94)	20 (87.0%)	5.70 ± 2.06 (4.74 – 6.66)	F=1.54	>0.05
Restriction in job activities		^2^	204 (100%)	3 [2–3]	92 (98.9%)	3 [2–3]	23 (100%)	2 [2–3]	H=1.15	>0.05
Restriction in leisure activities		^2^		3 [2–3]	93 (100%)	3 [2–4]		3 [2–3]	H=3.14	>0.05
Restriction in family activities		^2^		3 [2–3]	92 (98.9%)	3 [2–3]		3 [2–3]	H=1.57	>0.05
Surgery received due to dysmenorrhea		Yes (%)	194	155 (79.9 %)	85	68 (80.0 %) (0.71 – 0.89)	23	22 (95.7 %)	Fisher–Freeman–Halton exact	>0.05
Surgically confirmed endometriosis		0=no, 1=yes	144 (70.6%)	144 (70.6 %)	64 (68.8%)	64 (68.8 %)		22 (95.7 %)	χ² = 7.03	0.017*
Present intake of hormone treatment (contraceptive pill/endocrine dysmenorrhea/endometriosis treatment)										
Intake yes or no		Yes (%)	204	111 (54.4 %)	93	32 (34.4 %)	23	4 (17.4 %)	χ² = 18.4	<0.001*
Known estrogen-containing vs progestin-only		progestin-only (n)	111 (54.4 %)	84 (75.7 %)	32	21 (65.6 %)	4	2 (50 %)	Fisher’s exact	>0.05
Intake regimen	Continuous	Yes	111	90 (81.1%)	32	24 (75 %)	4	4 (100 %)	Fisher’s exact	>0.05
	With break/placebo interval			3 (2.7 %)		1 (3.1 %)		0 (0 %)	Fisher’s exact	>0.05
	IUD			18 (16.2 %)		7 (21.9 %)		0 (0 %)	Fisher’s exact	>0.05

Data include sociodemographics, migraine and menstrual pain characteristics, and the resulting treatment and restrictions in quality of life

Data are presented as mean ± SD (95 % CI) for continuous variables, median [IQR] for ordinal variables, and n ( %) for categorical variables

Statistical tests: Welch’s ANOVA for continuous variables, χ² or Fisher’s exact tests for categorical variables, and Kruskal–Wallis tests for ordinal variables

^1^0= no school leaving certificate, secondary school (Hauptschule) = 1, intermediate school (Mittlere Reife) = 2, high school (Abitur/Fachabitur) = 3, university degree = 4

^2^not at all = 0, slightly = 1, moderately = 2, severely = 3, very severely = 4

^3^no bleeding = 0, light bleeding = 1, moderate bleeding = 2, strong bleeding = 3

^4^no menstrual pain = 0, not disturbing = 1, marginally disturbing = 2, moderately disturbing = 3, severely disturbing = 4, very severely disturbing = 5

^5^0=never, 1=in less than 2 of 3 menstruations, 2=in two of three menstruations, 3=in every menstruation

*Significant results are highlighted in bold

The quantity of menstrual bleeding was higher in PM compared to RM or NM (*p* = 0.015; Table 3). All three groups showed high intensities of menstrual pain (mean VAS > 7) and a high disturbance level (mean disturbance level above 4 in all three groups, on a scale of 0 = no menstrual pain, 1 = not disturbing, 2 = marginally disturbing, 3 = moderately disturbing, 4 = severely disturbing, 5 = very severely disturbing; Table 3). Pain medication for menstrual pain reduced pain intensity only partially, with mean VAS levels remaining above 5 for all three groups; Table 3). Constraints in job, leisure and family activities due to menstrual pain also revealed mean restrictions ranging between moderate to severe intensity for all three groups (Table 3).

Participants with PM had the highest rate of surgically confirmed endometriosis (SCE) compared to those with RM or NM (*p* = 0.030; Table 3). However, the PM group had the lowest intake rate of hormone treatment (*p* < 0.001; Table 3).

**Fig. 2:**
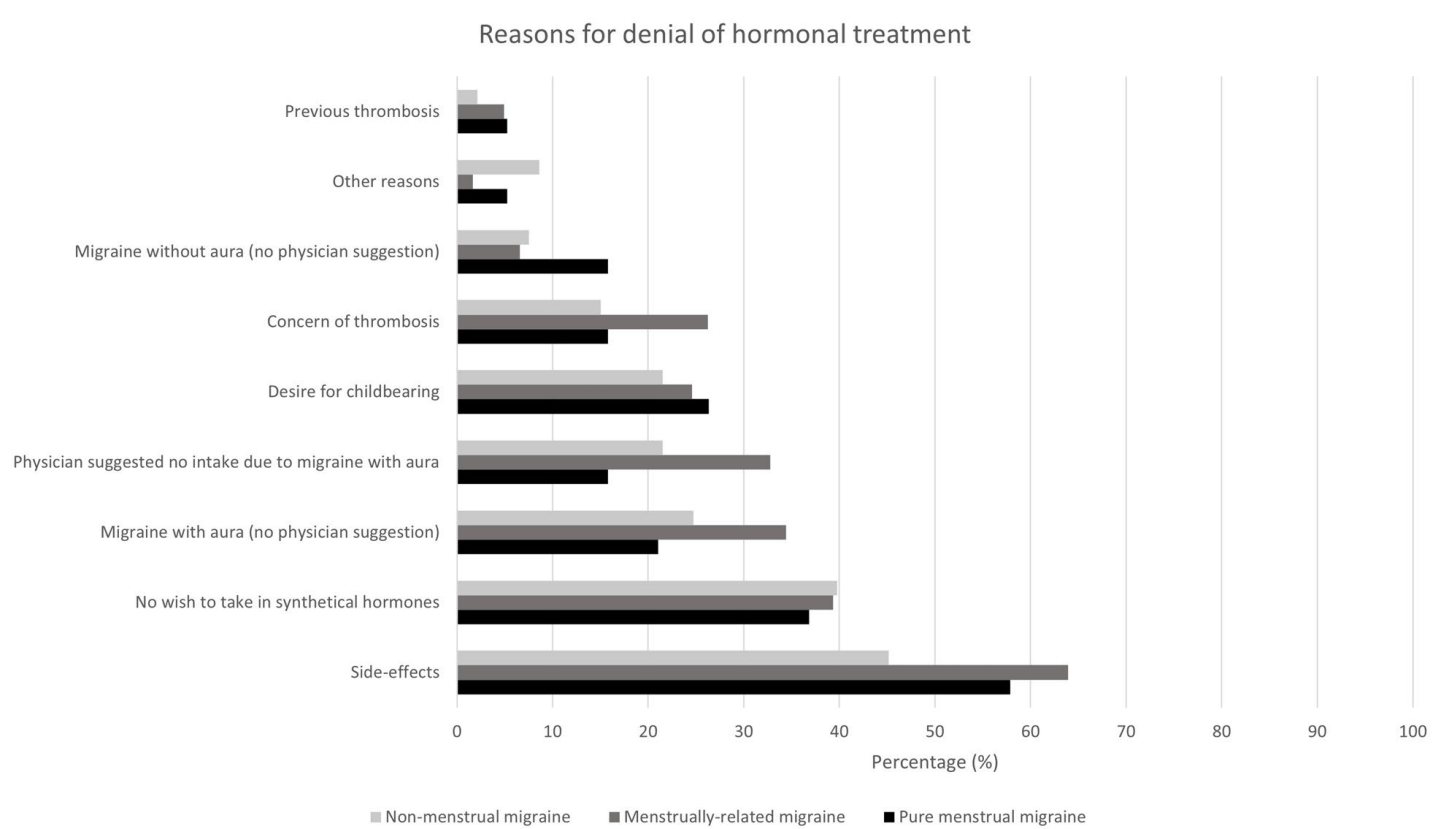
Reasons given by migraine participants for rejection of hormone treatment. Multiple answers were possible. There were no statistical differences between the three groups (NM, RM, PM)

Overall, 173 (54.1%) of migraine participants were not undergoing current hormone treatment. The most common reasons for the rejection of hormone treatment were side effects, a desire to avoid synthetic hormones, and migraine related concerns (for further details see Fig. 2). No significant differences between NM, RM, and PM were observed for these reasons (Fig. 2).

A detailed cross-classification of surgical status and current hormone therapy use is provided in Table 4. Overall, hormone therapy use did not significantly differ by surgical status among women with migraine. In the RM subgroup, however, surgical status was associated with current hormone therapy use (*p* = 0.049), with women with surgically confirmed endometriosis being more likely to use hormone therapy than those without surgical confirmation.

**Table 4:** Association between surgical status and current hormone therapy (HT) in women with and without migraine, stratified by migraine subtype

**Surgical status**	**No Migraine **		**Non-menstrual migraine **		**Menstrually-related migraine **		**Pure menstrual migraine **		**Any migraine **		**Total **	
	*n*=464		*n*=194		*n*=85		*n*=23		*n*=302		*n*=766	
	**HT: Yes**	**HT: No**	**HT: Yes**	**HT: No**	**HT: Yes**	**HT: No**	**HT: Yes**	**HT: No**	**HT: Yes**	**HT: No**	**HT: Yes**	**HT: No**
SCE	160 (34.5)	171 (36.9)	85 (43.8)	59 (30.4)	27 (31.8)	37 (43.5)	3 (13.0)	19 (82.6)	115 (38.1)	115 (38.1)	275 (35.9)	286 (37.3)
non-SCE (with prior surgery)	13 (2.8)	18 (3.9)	6 (3.1)	5 (2.6)	1 (1.2)	3 (3.5)	0 (0.0)	0 (0.0)	7 (2.3)	8 (2.6)	20 (2.6)	26 (3.4)
non-SCE (no prior surgery)	34 (7.3)	68 (14.7)	18 (9.3)	21 (10.8)	2 (2.4)	15 (17.6)	1 (4.3)	0 (0.0)	21 (7.0)	36 (11.9)	55 (7.2)	104 (13.6)
Test / statistic	χ² = 7.24		χ² = 2.07		Fisher–Freeman–Halton		Fishers exact		χ² = 3.18		χ² = 10.54	
*p*-value	0.027*		>0.05		0.049*		>0.05		>0.05		0.005*	

Data are presented as n (%)

Statistical tests: Pearson’s χ² or Fisher’s exact tests (Fisher–Freeman–Halton Monte Carlo for sparse tables), as appropriate

Significant results (*p* < 0.05) are shown in bold; trends (0.05 ≤ *p* < 0.10) in italics

A total of 766 participants (91.4 %) had complete data on surgical status, current hormone therapy, and migraine classification and were included in this analysis

*Significant results are highlighted in bold

## Discussion

To our knowledge, the present study is the first to provide a detailed differentiation of distinct migraine subtypes in endometriosis participants, including: (a) migraine with and without aura and (b) non-menstrual migraine (NM), menstrually-related migraine (RM) and pure menstrual migraine (PM). The investigation yielded very specific information about the characteristics of migraine and menstrual pain, as well as surgical and hormone treatment for endometriosis and/or dysmenorrhea.

### Co-occurrence of migraine and endometriosis

Our data showed that the association with migraine in general, as well as migraine with aura and pure menstrual migraine in particular, is higher in participants with surgically confirmed endometriosis compared to those with unconfirmed endometriosis. This could be related to pain and inflammatory pathways common to both chronic pain conditions, or an altered pain sensitivity in participants affected by both diseases [15–19]. There is strong evidence in the published literature to support the frequent co-occurrence of migraine and endometriosis [3, 13, 14, 20, 21]. The exact cause of this high co-occurrence is not fully understood. It is known that the neuropeptide CGRP plays an important role in migraine attacks, but whether it is involved in endometriosis remains inconclusive [22, 23]. Yet other authors have suggested common genetic determinants, but no causative gene has been found so far [24]. Only one genome-wide association study identified two common genetic loci in protein-coding genes (SLC35G6, TRIM32), which require further investigation [22, 23]. The co-occurrence of dysmenorrhea and migraine may also lead to a higher probability for surgery and subsequent diagnosis and treatment of endometriosis.

The diagnosis of both migraine [25] and endometriosis [26] is typically delayed by several years, leading to chronic pain and contributing the high levels of restrictions in job, leisure and family activities as observed in our study. In our cohort of women with surgically confirmed endometriosis, those with comorbid migraine reported higher dysmenorrhea intensity, heavier menstrual bleeding and greater restrictions in leisure and family life than women with endometriosis alone. However, the absolute differences in bleeding scores and dysmenorrhea intensity were small, and their clinical relevance remains uncertain. This is in line with findings from Selntigia et al., who reported that women with both endometriosis and migraine experience more intense and prolonged menstrual and pelvic pain as well as broader symptomatology compared with women with endometriosis alone [27].

Early diagnosis and greater awareness of both diseases and their co-occurrence are therefore crucial in improving the clinical management of both conditions. Participants with endometriosis should be screened for migraine and vice versa, and a multidisciplinary approach is needed to improve overall health outcomes as well as reduce the immense economic burden, including health care costs and productivity loss [28–30]. The co-occurrence of both diseases is known to involve more frequent absences from work and a reduction of work effectiveness [20].

Our sociodemographic analysis revealed statistical differences in BMI and age when comparing NM, RM and PM. The diagnosis of pure menstrual migraine appears to be more common in middle-aged women, while participants with non-menstrual migraine tend to be of younger average age [10]. A correlation has also been noted between a high BMI and a more frequent incidence of menstrual migraine attacks [10]. These data are in line with our observations. The published literature suggests that more severe forms of endometriosis are associated with a higher BMI [31]. This could well explain the highest BMI and the highest rate of SCE in the PM group.

### Migraine subtypes and endometriosis

**Fig. 3:**
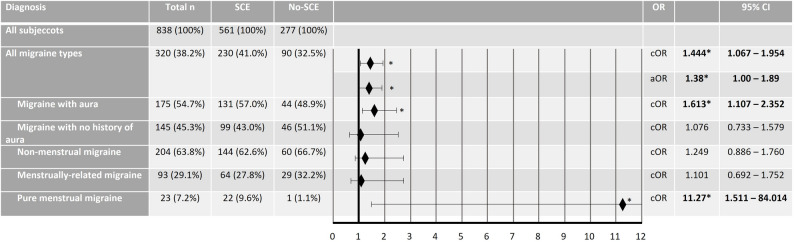
Odds Ratio of having pure menstrual migraine, menstrually-related migraine or non-menstrual migraine as a side diagnosis to surgically confirmed endometriosis (SCE) or dysmenorrhea, but no surgically-confirmed SCE (non-SCE). Presented are crude Odds Ratios (cOR) and adjusted Odds Ratio (aOR) for migraine overall. Significant results are highlighted*

Several studies have confirmed a higher odds of migraine diagnosis in participants with endometriosis [14, 15, 17, 20, 21, 27, 32–36]. However, recent meta-analytic evidence indicates that this association is not uniform across migraine subtypes: while endometriosis is significantly associated with migraine without aura, the evidence regarding migraine with aura remains highly uncertain, as reflected by the very wide confidence interval of the pooled estimate (OR 3.47, 95% CI 0.53–22.89) [14]. Our finding of an increased likelihood of migraine with aura in SCE compared to non-SCE (OR 1.61, 95%CI 1.11–2.35; Fig. 3) lies within this broad range.

Our study also revealed that the odds of pure menstrual migraine (PM) are higher in persons with SCE compared to non-SCE (OR 11.27, 95%CI 1.51–84.01; Fig. 3). This co-occurrence has received limited attention and has not been addressed so far. The wide confidence interval observed in our analysis reflects considerable statistical imprecision due to the small PM subgroup, and this finding should therefore be interpreted with caution. These restults are exploratory and hypothesis-generating in nature. While they do not allow causal conclusions, they point to a potentially relevant subgroup that warrants further investigation in prospective studies. Among migraine subtypes, RM showed the highest attack frequency and the strongest functional impairment across work, leisure and family life, indicating that RM represents the most burdensome phenotype in women with endometriosis. This is consistent with recent findings demonstrating that menstrual migraine is associated with a higher disease burden and reduced quality of life in endometriosis patients. A different study evaluated the prevalence of “menstrual migraine” in comparison with non-menstrual migraine in endometriosis, showing no statistical difference between groups [35]. However, these data should be viewed with caution because of the relatively small case numbers, and because menstrual migraine was not defined in accordance with the International Classification of Headache Disorders [9].

Hormone treatment suppressing menstruation may be effective in treating women with migraine, especially those with pure menstrual migraine [11, 37]. Cycle inhibition has been shown to reduce migraine attacks and migraine days, as observed with extended or continuous use of combined oral contraceptives or progestogen-only regimens [11, 37, 38], whereas cyclic regimens with hormone-free intervals are associated with an increase in attacks during the hormone-free days [39]. Extended-cycle oral hormone therapy has likewise been demonstrated to reduce menstrual headaches compared with placebo [40]. Despite this, our data showed that participants with PM received significantly less hormone treatment. Women with surgically confirmed endometriosis in our cohort were significantly more likely to use a continuous regimen, which aligns with the therapeutic aim of reducing menstruation-related symptoms. Continuous intake may be particularly relevant given that hormone-free intervals can precipitate migraine attacks, whereas cycle suppression reduces attack frequency, underscoring the need for coordinated endocrine and migraine management. We also observed that RM participants and women without migraine were more likely to use hormone therapy when endometriosis had been surgically confirmed, whereas no significant association was found for NM or PM. The most frequently cited reasons for the rejection of hormone treatment were side effects and the aversion to synthetic hormones. “Pill fatigue” (denial of hormone intake) and the decreasing use of hormonal contraceptives is a well known phenomenon in women and endometriosis participants [41–44]. A rising distrust, fostered by negative attitudes towards the combined oral contraceptive pill (COC) in social media, would explain the decline in the use of COC among young women in Western Europe [45]. The participants frequently mentioned their rejection of hormone treatment for endometriosis/dysmenorrhea, especially due to the diagnosis of migraine. This objection is worthy of discussion, as it is possible to treat dysmenorrhea and/or endometriosis with progestogen-only contraceptives, such as dienogest alone, without increasing the likelihood of thrombosis and/or stroke [46]. Prospective clinical data suggest comparable pain-related outcomes across different endocrine treatment regimens for endometriosis, underscoring the role of individualized long-term management [47]. Further possible hormonal treatment options (also in migraine participants) have been extensively discussed by Cirkel et al. [48]. Our data suggest that these participants are ineffectively treated and clearly require more intensive medical attention to improve their quality of life and alleviate their symptoms.

### Strengths and limitations

One of the key strengths of the present study is that it is the first to systemically evaluate migraine subtypes, while also providing a detailed analysis of migraine and menstrual pain characteristics. Data were collected via an online questionnaire. This method is widely recognized as a reliable and user-friendly tool for screening migraine participants [49, 50]. A further strength is the relatively large sample size, allowing for an in-depth analysis of a patient group that has not been studied in such detail before.

Nevertheless, some studies suggest potential over-reporting of the association between migraine attacks and menstruation, which should be taken into account [9]. In the present study, migraine classification was based on self-reported questionnaire data, and no prospective headache diaries were used. Although headache diaries are not mandatory for clinical diagnosis, their absence in a research setting may limit diagnostic precision. Accordingly, some degree of misclassification—particularly between RM and PM —cannot be excluded and should be considered when interpreting subtype-specific findings. Additionally, the representativeness of our cohort is worthy of attention. Our sample did not constitute a randomly selected population with matched controls, but mainly consisted of Caucasian middle-aged women who had received prior treatment at endometriosis centers or were recruited through the German Endometriosis Association. This recruitment approach may introduce selection bias and could limit the generalizability of the findings, as women with more severe symptoms or greater healthcare engagement may be overrepresented. A further limitation of our study is the potential misclassification within the non-SCE group. As only surgical confirmation was used for classification, participants without previous surgery — some of whom may have had sonographic signs — were assigned to the non-SCE group. Although surgically confirmed endometriosis could reflect a more advanced disease stage, we did not observe marked differences in menstrual pain intensity or functional impairment between surgically confirmed and non-surgically confirmed participants. Nevertheless, this classification approach may have led to some degree of diagnostic misclassification, potentially influencing effect estimates. As this is a cross-sectional study, causal or predictive relationships between migraine and endometriosis cannot be established, and this question should therefore be addressed in future longitudinal research. Given the exploratory nature of this study and the absence of formal adjustment for multiple comparisons, an increased risk of Type I error cannot be excluded. Accordingly, all findings should be interpreted as associations and hypothesis-generating rather than confirmatory.

## Conclusion

This study demonstrates a higher prevalence of migraine — particularly migraine with aura and pure menstrual migraine — in women with surgically confirmed endometriosis. Pure menstrual migraine showed the most pronounced association, a finding that has received limited attention in previous research. As PM is mechanistically linked to menstruation-related hormonal changes, cycle suppression represents a plausible management strategy. Our findings highlight the need to better understand treatment patterns and outcomes in this specific subgroup. Given the strong comorbidity of migraine and endometriosis, routine bidirectional screening and multidisciplinary management may enhance early diagnosis and patient outcomes.

## Supplementary Information

Supplementary Material 1.

## Data Availability

The datasets used and/or analyzed during the current study are available from the corresponding author on reasonable request.

## Abbreviations

ANOVA: Analysis of variance
BMI: Body mass index
CI: Confidence interval
GnRH: Gonadotropin–releasing hormone
ICHD3: International classification of headache disorders
MA: Migraine with aura
MO: Migraine participants who have never experienced migraine aura
NM: Non–menstrual migraine
Non-SCE: Participants with dysmenorrhea, but without surgically confirmed endometriosis (no surgery performed or surgery was performed but without evidence of endometriosis lesions)
OR: Odds ratio
PM: Pure menstrual migraine
RM: Menstrually–related migraine
SCE: Surgically confirmed endometriosis
SD: Standard deviation
VAS: Visual analog scale
