# Supplementary material for: Prevalence of migraine subtypes in women with endometriosis and/or dysmenorrhea: results from a cross-sectional multicenter trial
Source: BMC Womens Health. 2026 Feb 13;26:132. doi: 10.1186/s12905-026-04335-z (PMC12930695; doi:10.1186/s12905-026-04335-z)
Supplement: Supplementary file 1 — Supplementary Material 1. [file 12905_2026_4335_MOESM1_ESM.docx]

English translation of the parts/questions of the German Online-Questionnaire, which have been analyzed:

Dear prospective study participants,

are you female, suffering from menstrual pain or endometriosis, and are between menarche and menopause?

Then you are cordially invited to participate in this study.

Menstrual pain/endometriosis symptoms and headaches/migraines are common complaints that can affect women's quality of life. Women often suffer from both symptoms, sometimes several times during a month and sometimes simultaneously.

The extent to which these sometimes influence each other has been little researched.

In this study, we are investigating how many women experience period pain and headaches, how these are related, and how they may affect the other treatment. You are therefore explicitly invited to participate in this study even if you only have menstrual pain and no headaches.

The following questions should take no longer than 20 minutes to answer. The survey is anonymous. Therefore, it is not possible to identify you personally. The University of Lübeck's Ethics Committee has approved the conduct of this study.

Please note that due to the anonymous storage of data, withdrawal of participation and deletion of data is not possible.

If you have any questions about this study please contact us by email:

Principal investigator Dr. Christoph Cirkel and PD Dr. Anna Cirkel

Ratzeburger Allee 160

23538 Lübeck

[info.endokopf.luebeck@uksh.de](mailto:info.endokopf.luebeck@uksh.de)

Thank you and kind regards

- I want to participate in this trial and I consent to anonymous data storage
  - Yes
  - No

Thank you for wanting to take part in the study. The following questions are about your menstrual bleeding/menstrual pain. Please answer the questions spontaneously to the best of your knowledge. There are no "right" or "wrong" answers. Try to answer all questions completely.

- How would you rate the intensity of your menstrual bleeding?
  - strong bleeding
  - moderate bleeding
  - light bleeding
  - no bleeding
- Do you or have you had menstrual pain?
  - Yes
  - No
- How would you rate your menstrual pain over the last 3 months on a scale of 0-10? (0 = no pain to 10 = worst pain imaginable)


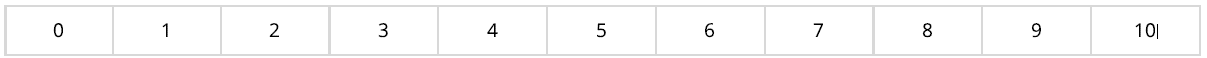


- How disturbing do you think your menstrual pain is?
  - I don’t have menstrual pain
  - not disturbing
  - marginally disturbing
  - moderately disturbing
  - severely disturbing
  - very severely disturbing
- Do you take pain medication for menstrual pain? (multiple answers possible)
  - No, I don’t take pain medication
  - Yes, ibuprofen
  - Yes, aspirin
  - Yes, naproxen
  - Yes, paracetamol
  - Yes, buscopan
  - Yes, metamizole
  - Yes, I take the following pain medication: …
- How would you rate your menstrual pain over the past 3 months on a scale of 0-10 while taking pain medication? (0 = no pain to 10 = worst pain imaginable)


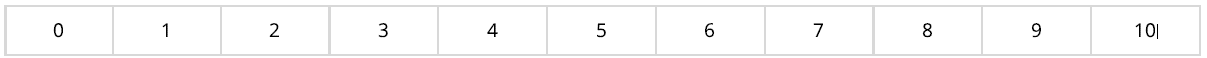


- How often do you take pain medication (ibuprofen, paracetamol, etc.) for menstrual pain (on average)?
  - I don’t need pain medication
  - I need pain medication in less than 2 out of 3 cycles
  - I need pain medication in 2 out of 3 cycles
  - I need pain medication every cycle
- Are you taking a hormonal medication (e.g. hormonal contraceptives or similar) due to menstrual pain or endometriosis?
  - Yes
  - No
  - Unknown
- How would you rate your menstrual pain over the last 3 months on a scale of 0-10 while taking hormone medications (e.g., hormonal contraceptives or similar)? (0 = no pain to 10 = worst pain imaginable)


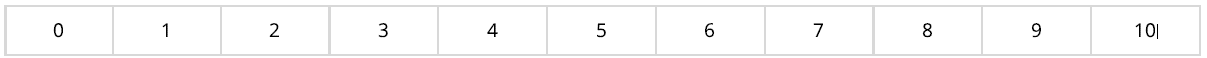


- Are you currently taking the pill or hormonal endometriosis therapy, or are you using other hormonal contraceptives?
  - No, I do not use hormonal contraception or take any hormone-based contraceptives.
  - Yes, I take dienogest in a long-term cycle.
  - Yes, I take a contraceptive pill for 21 days followed by a break or tablets without active ingredients (placebo)
  - Yes, I take a contraceptive pill in a long-term cycle with a planned break every few months
  - Yes, I take a contraceptive pill (mini-pill; progestogen-only preparation) continuously without a break.
  - Yes, I take contraceptive pill continuously without a break, but I cannot state whether it is a combined preparation or a progestogen only pill.
  - Yes, I have a hormonal coil (hormonal IUD) inserted
- What is the name of your contraceptive pill/hormone preparation?
  - Unknown
  - Name of the pill: …
- Have you not been prescribed a hormonal medication (the pill) because of migraine with aura?
  - Yes
  - No
  - Not applicable/unknown
- Have you been recommended a hormone preparation (pill) due to menstrual pain or endometriosis, but you are not taking or have not taken it? (multiple answers possible)
  - No, this is not the case for me
  - Yes, due to the desire to have children.
  - Yes, I don't take them because of side effects.
  - Yes, I would like to live without artificially administered hormones.
  - Yes, due to a migraine with aura.
  - Yes, due to migraine without aura
  - Yes, I am worried about thrombosis
  - Yes, I have had thrombosis
  - Other reasons: …
- How much are you restricted in your job activities by menstrual pain?
  - Not at all
  - Slightly
  - Moderately
  - Severely
  - Very severely
- How much are you restricted in your leisure activities by menstrual pain?
  - Not at all
  - Slightly
  - Moderately
  - Severely
  - Very severely
- How much are you restricted in your family activities by menstrual pain?
  - Not at all
  - Slightly
  - Moderately
  - Severely
  - Very severely
- Have you been diagnosed with or suspected of endometriosis, or have you sought medical treatment based on your (suspected) diagnosis of endometriosis?
  - Yes
  - No
  - I don’t know what endometriosis is.

You have now answered the first part of the questionnaire. Thank you very much

The following questions are about headaches. Please answer the questions spontaneously to the best of your knowledge. There are no "right" or "wrong" answers. Try to answer all questions completely

- Do you suffer from headaches? (multiple answers possible)
  - No, I don’t have a headache and I haven’t had any before
  - Yes, I have migraine
  - Yes, I have migraine with aura
  - Yes, I have tension-type headache
  - Yes, I have cluster headaches
  - Yes, I have medication overuse headache
  - Yes, but I am not familiar with the type of headache
  - Yes, I have other headaches. Please enter their diagnosis here: …
- Do you have migraines several times a month or less frequently?
  - I have several migraine attacks a month
  - I only have migraine attacks every few months
- How often do you have migraines per month (on average)?
  - Please state: …
- On a scale of 0-10, how severe is your migraine on average? (from 0= no pain to 10= strongest imaginable pain)


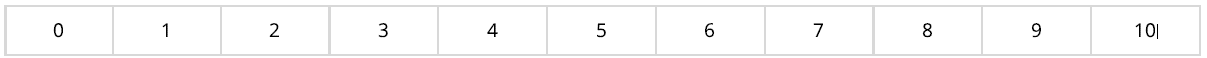


- Do you take pain medication for the headache? (multiple answers possible)
  - No
  - Ibuprofen
  - Metamizole
  - Aspirin
  - Triptans (e.g. Sumatriptan, Naratriptan, Rizatriptan, ...)
  - Botox injections
  - Pain prophylaxis (e.g. beta-blockers, topiramate, amitriptyline, ...)
  - Antibodies (e.g. erenumab, galcanezumab, fremanezumab)
  - female sex hormones (e.g. contraceptive pill)
  - Naproxen
  - Paracetamol
  - Unknown
  - Other medication: …
- If you have taken painkillers for migraines: On how many days per month in which you suffer from migraines (on average)? (If you are not taking any medication for this, enter the number "0")
  - Please state: …
- How much are you restricted in your job activities due to migraines?
  - Not at all
  - Slightly
  - Moderately
  - Severely
  - Very severely
- How much are you restricted in your leisure activities due to migraines?
  - Not at all
  - Slightly
  - Moderately
  - Severely
  - Very severely
- How much are you restricted in your family activities due to migraines?
  - Not at all
  - Slightly
  - Moderately
  - Severely
  - Very severely
- Do you notice a temporal connection between the migraine and your menstruation? (Temporal connection= the headaches 2 days before the menstruation up to 3 days after the menstruation)
  - No, I don't notice any temporal connection
  - Yes, with less than 2 out of 3 menstruations there is a temporal connection
  - Yes, there is a temporal connection in at least 2 out of 3 menstruations
- If there is a temporal connection between the migraine and your menstruation: Is this always the case? ((Temporal connection= the headaches 2 days before the menstruation up to 3 days after the menstruation)
  - Yes, the migraine occurs exclusively in connection with the menstruation and at no other time
  - No, I (also) have migraines without a temporal connection to my menstruation

Thank you very much. You have now answered the second part of the survey.

In the following questions we would like to know more about your (suspected) diagnosis of endometriosis.

Please answer the questions spontaneously to the best of your knowledge. There are no "right" or "wrong" answers. Try to answer all questions completely.

- Have you had one or more laparoscopies to diagnose or treat endometriosis or menstrual pain?
  - Yes, one surgery
  - Yes, two surgeries
  - Yes, three surgeries
  - Yes, four surgeries
  - Yes, more than four surgeries
  - No, I did not have a surgery
- What type of endometriosis do you have?
  - The diagnosis of endometriosis has not been confirmed / I do not have endometriosis
  - Peritoneal endometriosis
  - Ovarian Endometriosis
  - Uterine endometriosis / adenomyosis uteri
  - Endometriosis with bladder involvement
  - Endometriosis with bowel involvement
  - Rectovaginal endometriosis
  - Deeply infiltration endometriosis of the pelvic sidewall
  - Scar endometriosis
  - Other endometriosis
  - Unknown/ not specified
- Were endometriosis lesions found during the surgery/surgeries and were these also removed?
  - Yes, I had endometriosis lesions found and they were all removed.
  - Yes, endometriosis lesions were found, but not all of them were removed
  - No, no endometriosis lesions were found.
  - Yes, endometriosis lesions were found, but I don't know whether they were all removed.
  - I don't know whether endometriosis lesions were found

You have now completed this part of the survey. Thank you very much.

Now we come to the last part. Please try to answer all questions completely.

- How old are you? (years)
  - Please specify: …
- How tall are you? (cm)
  - Please specify: …
- How much do you weigh? (kg)
  - Please specify: …
- Are you in menopause?
  - Yes
  - No
- What school or university degree do you have (highest degree)?
  - no school leaving certificate
  - secondary school (Hauptschule)
  - intermediate school (Mittlere Reife)
  - high school (Abitur/Fachabitur)
  - university degree
